# The Maize AAA-Type Protein SKD1 Confers Enhanced Salt and Drought Stress Tolerance in Transgenic Tobacco by Interacting with Lyst-Interacting Protein 5

**DOI:** 10.1371/journal.pone.0069787

**Published:** 2013-07-24

**Authors:** Zongliang Xia, Yangyang Wei, Kaile Sun, Jianyu Wu, Yongxia Wang, Ke Wu

**Affiliations:** 1 College of Life Science, Henan Agricultural University, Zhengzhou, PR China; 2 Key Laboratory of Physiology, Ecology and Genetic Improvement of Food Crops in Henan Province, Zhengzhou, PR China; Iowa State University, United States of America

## Abstract

ATPase associated with various cellular activities (AAA) proteins are important regulators involved in diverse cellular functions. To date, the molecular mechanisms of AAA proteins involved in response to salt and drought stresses in plants are largely unknown. In this study, a putative *SKD1* (suppressor of K ^+^ transport growth defect 1) ortholog from *Zea mays* (*ZmSKD1*), which encodes a putative AAA protein, was isolated. The transcript levels of *ZmSKD1* were higher in aerial tissues and were markedly up-regulated by salt or drought stress. Over-expression of *ZmSKD1* in tobacco plants enhanced their tolerances not only to salt but to drought. Moreover, reactive oxygen species accumulations in *ZmSKD1* transgenic lines were relative less than those in wild-type plants during salt or PEG-induced water stress. The interaction between ZmSKD1 and NtLIP5 (Lyst-Interacting Protein 5 homolog from *Nicotiana tabacum*) was confirmed by both yeast two-hybrid and immuno-precipitation assays; moreover, the α-helix-rich domain in the C-terminus of ZmSKD1 was identified to be required for its interaction with NtLIP5 using truncation mutations. Collectively, these data demonstrate that ZmSKD1could be involved in salt and drought stress responses and its over-expression enhances salt or drought stress tolerance possibly through interacting with LIP5 in tobacco. This study may facilitate our understandings of the biological roles of SKD1-mediated ESCRT pathway under stress conditions in higher plants and accelerate genetic improvement of crop plants tolerant to environmental stresses.

## Introduction

ATPase associated with various cellular activities (AAA) proteins constitute a family of highly conserved proteins present in all organisms. They are important regulators involved in diverse cellular functions, including vesicle trafficking, proteosome-mediated protein degradation, and chaperone-like activity [1], [2], [3].

SKD1 (suppressor of K ^+^ transport growth defect 1), a member of AAA family, has been identified from yeast, mammals and plants [4]–[8]. Mammalian *SKD1* was first identified by screening mouse cDNA library for complementing the K^+^-uptake-defect phenotype in yeast *vps4* mutant [4]. The structure of SKD1 consists of a central AAA domain capable of catalyzing ATP hydrolysis and an N-terminal MIT (microtubule interacting and trafficking) domain. Mammalian *SKD1* and its yeast homolog *VPS4* (vacuolar protein sorting 4) are orthologues and involved in intracellular protein trafficking [6]. SKD1 participates in the sorting of monoubiquitylated transmembrane cargo to the lysosome/vacuole by disassembling the members of the ESCRT (endosomal sorting complex required for transport) complexes from the endosomal membrane [5], [9], [10], [11]. More importantly, yeast mutants defective in VPS4 display a salt-sensitive phenotype, suggesting a strong link between ESCRT-mediated endosomal/vacuolar trafficking and salt tolerance [11].

In higher plants, *SKD1* homolog from the halophyte ice plant *Mesembryanthemum crystallinum* (*mcSKD1*) was first identified as a salt-induced gene by suppression subtractive hybridization [12] and was found capable of complementing the phenotype of yeast *vps4* mutant [7].The *Arabidopsis SKD1* homolog (*AtSKD1*) has been shown to be an ortholog of SKD1/Vps4 with ATPase activity and colocalize with endosomal markers on multivescular bodies (MVBs) [8]. However, overexpression of dominant-negative AtSKD1^E232Q^ is lethal for *Arabidopsis* plants [8]. Further studies have showed that AtSKD1 is involved in MVB function and contributes to vacuolar maintenance; moreover, AtSKD1 can interact with its positive regulator LYST-INTERACTING PROTEIN5 (LIP5) in yeast cells [8], [13]. Most recently, direct genetic evidence has shown that reduced expression of *AtSKD1* decreases salt tolerance in *Arabidopsis* using RNA interference approach, suggesting *AtSKD1* participates in salt tolerance in plants [14].

In spite of the progress made in understanding molecular and biological function of *SKD1* in model plant, the knowledge of molecular and functional aspects of the *SKD1* from higher plants is still limited. Maize (*Zea mays*) is an important cereal crop worldwide that is a staple food to many populations. Salt and drought stresses often adversely affect plant growth and productivity, thus they are becoming serious problems in a plenty of maize-planting regions worldwide. Unfortunately, the molecular mechanisms of AAA proteins involved in response to salt and drought stresses in plants are largely unknown, let alone in crop plants. In this study, a putative *SKD1* ortholog from *Zea mays* (*ZmSKD1*), which encodes a putative AAA-type protein, was found by a salt-induced microarray analysis. We further characterized the putative maize *SKD1* homolog in transgenic tobacco to investigate salt and drought tolerances and possible function mechanisms.

## Results

### Molecular Characterization of *ZmSKD1*

**Figure 1:**
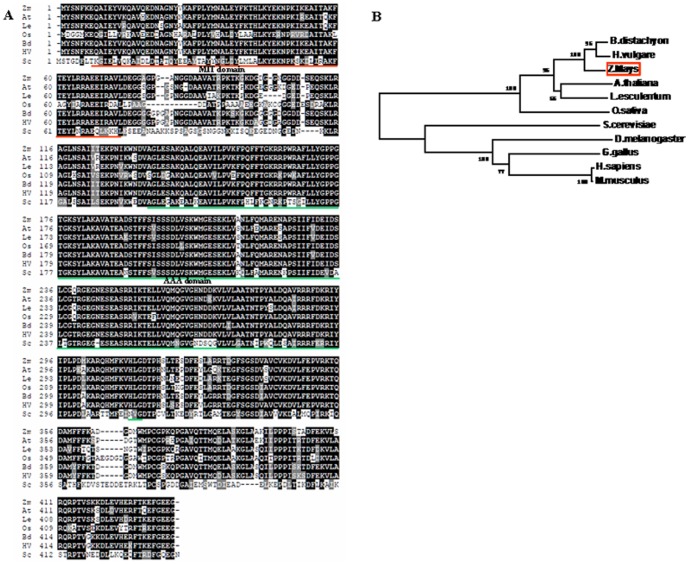
Sequence alignment and phylogenetic analysis of SKD1 proteins from *Zea Mays* and other species. **A** An alignment is shown for the deduced amino acid sequence of SKD1s from *Zea Mays* (Zm), *Arabidopsis thaliana* (At), *Lycopersicon esculentum* (Le), *Oryza sativa* (Os), *Brachypodium distachyon* (Bd), *Hordeum vulgare* (Hv) and *Saccharomyces cerevisiae* (Sc). The numbers on the left indicate the amino acid position. Identical residues in all these proteins are shown in a black background. Dashes indicated gaps introduced for optimal alignment. The putative MIT and AAA domains are underlined with a thick red line and a thick green line, respectively. **B** Phylogenetic tree based on SKD1 protein sequences from yeast, plants, and animals. The bootstrap values shown were calculated based on 500 replications. The tree was constructed using the neighbor-joining method. *Z.mays*, AY105155; *A.thaliana*, At2g27600; *L.esculentum*, AK324437; *O.sativa*, AF499028; *B.distachyon*, LOC100837561; *H.vulgare*, AK359160; *D.melanogaster*, NP_573258; *G.gallus*, AJ720732; H.sapiens, AF038960; *M.musculus*, NP_033216; *S.cerevisiae*, NP_015499.

At the start of this work, total RNAs from the leaves of maize plants, which had been treated with 200 mM NaCl, were used as samples for microarray experiments. A partial cDNA fragment with about 12-fold induction was obtained (data not shown). Homology search by BLAST analysis showed that the gene is highly homologous to *AtSKD1*, a member of the AAA-type ATPase family in *Arabidopsis*, and thus is named *ZmSKD1* (accession no. AY105155).The ORF of the *ZmSKD1* consists of 1308 nucleotide acids and encodes a protein of 435 amino acids with a predicted molecular mass of about 48 kDa. Like other known plant SKD1s, the deduced amino acid sequence of the ZmSKD1 exhibits similar structural characteristics with two functional domains. One is a central AAA domain (residues 135 to 298); the other is an N-terminal MIT domain (residues 7 to 73) (Fig. 1A). Amino acid sequence comparisons have revealed that ZmSKD1 exhibits high identity to counterpart proteins from *Arabidopsis thaliana* (86% identity), *Lycopersicon esculentum* (87% identity), *Oryza sativa* (74% identity), *Brachypodium distachyon* (92% identity) and *Hordeum vulgare* (93% identity), but low identity to *Saccharomyces cerevisiae* (52% identity) (Fig. 1A).

A phylogenetic tree was established based on SKD1 protein sequences available in GenBank from 12 species including 6 plant species (*Arabidopsis*, tomato, rice, barley, wild wheat and maize), 5 animal species (human, mouse, drosophila, nematode, chicken), and *S.cerevisiae* (Fig. 1B). As shown in Fig. 1B, these SKD1s were clustered into two distinct groups, including plant SKD1 and animal SKD1 groups. In plant SKD1s, interestingly, SKD1 from rice (OsSKD1) formed a subgroup distinct from the other SKD1s subgroup, including the monocot SKD1s (HvSKD1, BdSKD1 and ZmSO) and the dicot SKD1s (AtSKD1 and LeSKD1). The ZmSKD1 showed higher identities with BdSKD1 or HvSKD1, and thus was clustered into the same isoform subgroup. The dicot SKD1s (AtSKD1 and LeSKD1) were clustered into the same isoform subgroup. On the other hand, in animal SKD1s, the *S.cerevisiae* SKD1 (ScSKD1) formed a subgroup distinct from the SKD1s from human (HmSKD1), mouse (MmSKD1), drosophila (DmSKD1), nematode (CeSKD1), and chicken (GgSKD1). The SKD1s from mammals (HmSKD1 and MmSKD1) were clustered into the same isoform subgroup. These results clearly demonstrated that ZmSKD1 shares basic structural feature similar to the known SKD1 proteins from yeast, *Arabidopsis* and mammals, thus could be identified as an ortholog of SKD1.

### Transcript Levels of *ZmSKD1* in Various Organs of Maize and Its Responses to Salt or PEG-induced Water Stress

**Figure 2:**
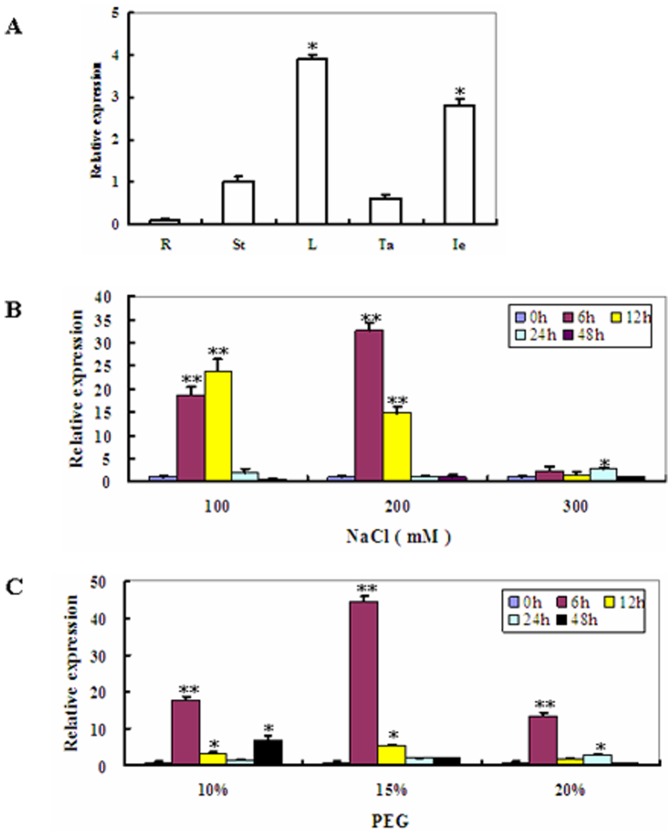
Transcript profiles of *ZmSKD1* in major organs of maize and its responses to salt and drought stresses. **A** The transcriptional pattern of *ZmSKD1* in maize root (R), stem (St), leaf (L), tassel (Ta) and immature ear (Ie) samples evaluated by real-time PCR. For each real-time PCR, the transcript levels of maize internal control gene *Ubiquitin* were also evaluated in various samples. For each assay, the expression level in stems was defined as 1.0, and data represented means ± SE of three technical replicates. *t-test, with P<0.05. **B** Time-course analysis of *ZmSKD1* transcript levels under various concentrations of salt treatments by real-time PCR. Two-week-old maize seedlings were exposed to 100, 200, and 300 mM NaCl for indicated time points (0, 6, 12, 24, and 48 h), and leaf samples were used for real-time PCR analysis. **C** Time-course analysis of *ZmSKD1* transcript levels under PEG-induced water stress by real-time PCR. Two-week-old maize seedlings were exposed to 0, 10%, 15%, and 20% PEG6000 for indicated time points (0, 6, 12, 24, and 48 h), and leaf samples were used for real-time PCR analysis. In both B and C assays, *Ubiquitin* was used as an internal control. For each treatment, the expression level at time point 0 was defined as 1.0, and data represented means ± SE of three technical replicates. **t-test, with P<0.01; *t-test, with P<0.05.

Transcriptional patterns of *ZmSKD1* were examined in five organs (roots, stems, leaves, tassels, and immature ears) by qRT-PCR. As shown in Fig. 2A, *ZmSKD1* mRNA was detected in roots, stems, leaves, tassels or immature ears. The *ZmSKD1* transcript levels were significantly high in leaves and immature ears. In contrast, *ZmSKD1* transcripts were low in roots, tassels and stems (Fig. 2A). The highest relative expression occurred in the leaves, with about 30 times as high as that of the roots (Fig. 2A).

Time-course analysis of *ZmSKD1* transcript levels in maize plants under salt or PEG-induced water stress was performed by qRT-PCR (Fig. 2B, 2C). Under salt treatment, *ZmSKD1* was significantly activated by 100 or 200 mM of NaCl (Fig. 2B). This trend was clearly seen at 6 and 12 h of the treatment period (Fig. 2B). Under PEG treatment, *ZmSKD1* was significantly activated by three concentrations of PEG (10%, 15%, and 20%) at 6 or 12 h of the treatment period with a peak at 6 h (17, 44, and 13-fold increases, respectively) (Fig. 2C). These results suggested that *ZmSKD1* was responsive not only to salt but to drought stress, and up-regulated significantly by these stresses at the early stage.

### Responses of *ZmSKD1*-overexpressing Tobacco Plants to Salt and Drought Stresses

**Figure 3:**
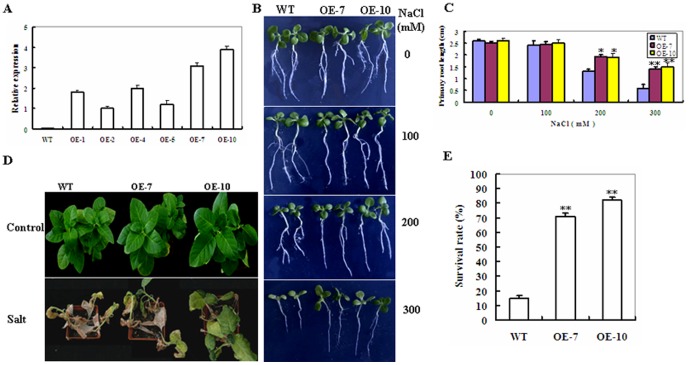
Phenotypes of wild-type and *ZmSKD1*-overexpressing tobacco plants in response to salt stress. **A** Transcription levels of *ZmSKD1* in wild-type (WT) tobacco plants and six homozygous over-expression (OE) lines (named OE-1, OE-2, OE-4, OE-5, OE-7 and OE-10). *ZmSKD1* transcripts detected by qPCR were present in the OE lines, but not in the wild-type plants. **B** Growth phenotypes of 2-week-old WT and transgenic OE (OE-7 and OE-10) seedlings vertically growing on 1/2 MS medium supplemented with 0, 100, 200 and 300 mM NaCl for 10 d. **C** Primary root length of 10 d-salt stressed plants in Fig. B. Values are mean ± SE, n = 10. **t-test, with P<0.01; *t-test, with P<0.05. **D** Representative phenotype of 6-week-old WT and transgenic OE plants growing in soil pots supplied with 0 (water) or 300 mM NaCl solution every other day for 4 weeks. **E** Survival rates (%) under salinity stress in Fig. D were determined as the number of visibly green plants after 4 weeks. Values are mean ± SE, n = 10. **t-test, with P<0.01.

To explore the physiological function of *ZmSKD1*, a CaMV 35S promoter-driven binary expression construct harboring *ZmSKD1* was developed and transformed into tobacco by *Agrobacterium*-mediated transformation. To this end, six homozygous transgenic lines (named OE-1, OE-2, OE-4, OE-5, OE-7 and OE-10) were developed, in which *ZmSKD1* transcript levels were analyzed by qRT-PCR (Fig. 3A). Both lines (OE-7 and OE-10) with higher *ZmSKD1* transcripts were chosen for further analysis.

To investigate whether overexpression of *ZmSKD1* in plants enhances salt tolerance, seedlings at different developmental stages from transgenic lines (OE-7 and OE-10) and WT were treated separately with different concentrations of NaCl. When 2-wk-old seedlings were transferred to medium supplemented with NaCl and grown for 10 d, the WT plants showed more chlorosis and stunted phenotypes than the OE lines under both 200 and 300 mM NaCl. This was clearly seen upon 300 mM NaCl exposure (Fig. 3B). No significant changes were observed in growth phenotypes between WT and OE plants under 0 and 100 mM of NaCl (Fig. 3B). Root length determinations showed that significant differences were observed between WT and OE plants under 200 or 300 mM of NaCl. Compared with unstressed seedlings, root length decreased by 50% for 200 mM NaCl and 77% for 300 mM NaCl, respectively in the WT plants. By contrast, root length in both OE lines only decreased by 24 and 27% for 200 mM NaCl and 44 and 42% for 300 mM NaCl, respectively (Fig. 3C). When 6-wk-old transgenic plants were exposed to a continuous stress consisting of 300 mM NaCl added to the soil every other day for 4 weeks. The growth of WT plants was severely affected and more than 80% of WT plants were dead, whereas 70–80% of transgenic plants were still alive (Fig. 3D and 3E). These results demonstrate that overexpression of *ZmSKD1* in transgenic tobacco plants significantly enhances tolerance to salt stress.

**Figure 4:**
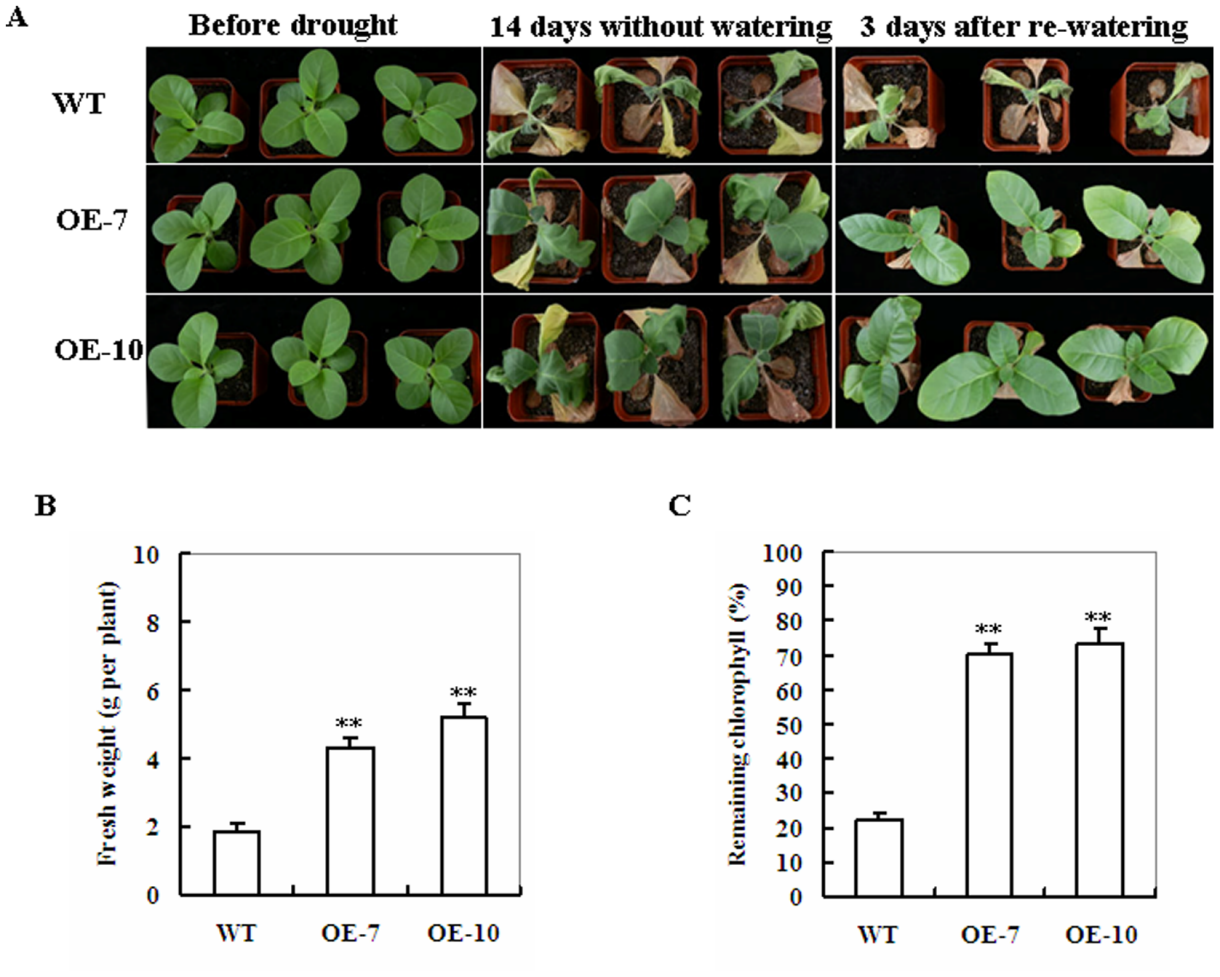
Phenotypes of wild-type and *ZmSKD1*-overexpressing tobacco plants in response to drought stress. **A** Drought tolerance of potted plants of wild-type and *ZmSKD1*-overexpressing tobacco. Four-week-old WT and transgenic OE (OE-7 and OE-10) plants were grown in soil in pots for 14 d without watering, and then rewatering after 3 days. **B** Fresh weight of drought-stressed wild-type and *ZmSKD1*-overexpressing plants after 3 days recovery. Values are mean ± SE, n = 6. **t-test, with P<0.01. **C** Relative remaining chlorophyll (%) of drought-stressed wild-type and *ZmSKD1*- overexpressing plants after 3 days recovery. Values are mean ± SE, n = 6. **t-test, with P<0.01.

To characterize the performance of *ZmSKD1* transgenic lines under watering-stress (drought) in soil, both OE lines were tested at the seedling stage (4-wk old). Under well-watered conditions, there was no obvious difference between WT and transgenic lines in leaves size and number of plants (Fig. 4A). After 14 days without watering, all WT plants displayed severe wilting (all leaves were severely curled and more than 70% leaves were turning yellow and dead), whereas *ZmSKD1* transgenic lines showed signs of moderate water stress and most upper leaves of transgenic plants were still green and fully expanded (Fig. 4A). Three days after rewatering, all WT plants were nearly dead, whereas all transgenic lines survived the stress and started to grow (Fig. 4A). Accordingly, plant biomass and remaining chlorophyll content in *ZmSKD1* transgenic lines were significantly higher than those in WT plants (2.5 and 3-fold on the average, respectively). (Fig. 4B, 4C). These results provide evidence that overexpression of *ZmSKD1* in transgenic tobacco plants also improves tolerance to drought stress.

### ROS Accumulations in *ZmSKD1* Transgenic Plants During Salt or Drought Stress

**Figure 5:**
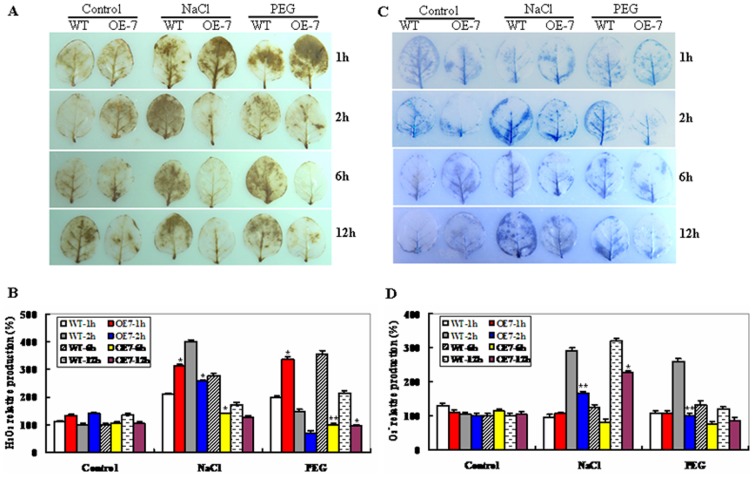
Dynamic changes of ROS in wild-type and *ZmSKD1*-overexpressing tobacco plants in response to salt or PEG-induced water stress. **A** H_2_O_2_ production in leaves of wild-type and OE (OE-7) plants treated with distilled water(control), 200 mM NaCl or 15% PEG-6000 solutions, respectively, was visualized by staining with 3, 3′-diaminobenzidine (DAB). Plants were treated for 1, 2, 6, and 12 h, and were subsequently stained with DAB as described in the experimental procedures. **B** Relative H_2_O_2_ levels were quantified in leaves of wild-type and OE (OE-7) plants exposed to 200 mM NaCl or 15% PEG-6000 for 1, 2, 6, and 12 h. Error bars indicate SE (n = 6). **t-test, with P<0.01; *t-test, with P<0.05. **C** O_2_^−^ production in leaves of wild-type and OE (OE-7) plants treated with 200 mM NaCl or 15% PEG-6000 solutions, respectively, was visualized by staining with nitroblue tetrazolium (NBT). Plants were treated for 1, 2, 6, and 12 h, and were subsequently stained with NBT as described in the experimental procedures. **D** Relative O_2_^−^ levels were quantified in leaves of wild-type and OE (OE-7) plants exposed to 200 mM NaCl or 15% PEG-6000 for 1, 2, 6, and 12 h. Error bars indicate SE (n = 6). **t-test, with P<0.01; *t-test, with P<0.05.

Drought or salt stress may induce production of reactive oxygen species (ROS), which cause oxidative damage to plant cells, or act as signaling molecules for regulating development and various physiological responses [15]. This promoted us to examine cellular levels of two prominent ROS (O_2_^−^ and H_2_O_2_) accumulations in OE and WT plants during salt or PEG-induced water stress. Histochemical detection of H_2_O_2_ accumulation was performed with DAB staining in NaCl or PEG-treated leaves along with controls from OE and WT plants within 12 h. As shown in Fig. 5A, both OE and WT leaves showed more DAB staining at 1 h of NaCl or PEG treatment; after 2 h, marked difference in DAB staining intensity was observed between OE and WT leaves. WT showed more DAB staining at 2 h of salt or drought stress, and remained higher levels till 12 h, whereas OE showed less staining after 2 h; moreover, the contrasting staining was much clear between the OE and WT leaves at 6 h, and till the end of the stresses (Fig. 5A). No significant differences in DAB staining intensity were observed between WT and OE lines within 12 h of distilled water-treated controls (Fig. 5A). The fluctuation in H_2_O_2_ level, especially in wild-type plants treated with PEG and stained with DAB was also noted (Fig. 5A). Quantitative determination of H_2_O_2_ accumulation at 1, 2, 6 and 12 h of salt or drought stress further demonstrated that compared to their corresponding controls, WT increased significantly in DAB staining intensity at 2, 6, or 12 h of both stresses, while the OE plants had no significant changes (Fig. 5B).

Similarly, O_2_^−^ accumulation was examined *in situ* with NBT staining. As shown in Fig. 5C, both OE and WT leaves showed more NBT staining at 2 h of NaCl or PEG treatment; after 2 h, marked difference in NBT staining intensity was observed between OE and WT leaves. WT showed increasing NBT staining at 2 h, and remained high levels till 12 h, whereas OE showed decreasing staining after 2 h; moreover, the contrasting staining was clear between the OE and WT leaves at 2 h, after that the difference is becoming unclear in the remaining period of the stresses (Fig. 5C). No significant differences in NBT staining intensity were observed between WT and OE lines within 12 h of distilled water-treated controls (Fig. 5C). Also, there is a fluctuation in O_2_^−^ level, especially in wild-type and the OE plants treated with salt and stained with NBT (Fig. 5C). Quantitative determination of O_2_^−^ accumulation at 1, 2, 6 and 12 h of salt or drought stress further showed that compared to their corresponding controls, WT increased significantly in DAB staining intensity at 2 h of both stresses, while the OE plants had no significant changes (Fig. 5D). These results demonstrate that ROS accumulations in *ZmSKD1* transgenic lines were relative less than those in WT plants during salt or PEG-induced water stress.

### ZmSKD1 Interacts with NtLIP5 in Yeast and *in planta*

In *Arabidopsis*, LIP5 (LYST-INTERACTING PROTEIN 5) has been characterized as an AtSKD1 interactor [8]. Therefore, it would be of interest to examine whether ZmSKD1 had the ability to interact with LIP5 homolog from tobacco (NtLIP5) in transgenic plants. *NtLIP5* was cloned by RT-PCR from tobacco leaves, which encodes a predicted protein of 414 amino acids. This protein is 59.4% identical to AtLIP5 (At4g26750). A database search revealed that there is only one *LIP5* gene in the released tobacco genome (Data not shown).

**Figure 6:**
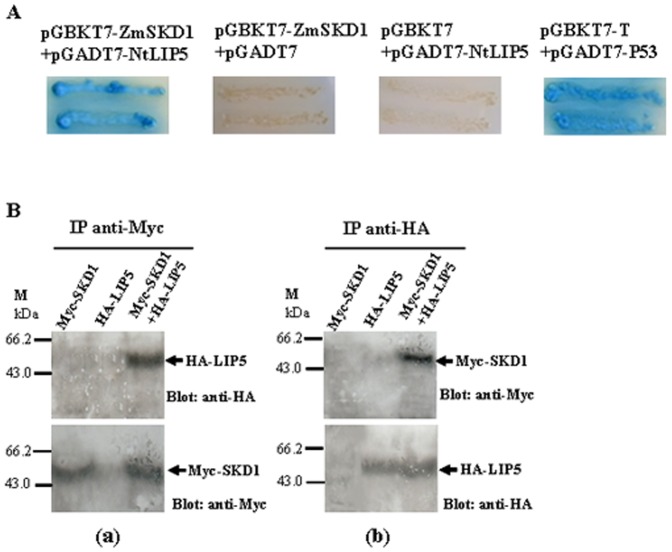
ZmSKD1 interacts with NtLIP5 in yeast and *in planta*. **A** ZmSKD1 interacts with NtLIP5 in YTH system. Yeast colonies containing pGBKT7-SKD1/pGADT7-LIP5 grew well on the selective medium (SD/−Ade/−His/−Leu/−Trp) and turned blue in the β-galactosidase assays as did yeast colonies containing pGBKT7-T/pGADT7-p53, which was used as the positive control, whereas yeast transformed with pGBKT7-SKD1/pGADT7 or pGBKT7/pGADT7-LIP5 used as negative controls were unable to grow. **B** ZmSKD1 interacts with NtLIP5 in coimmunoprecipitation assays in *Nicotiana benthamiana*. Different cell lysates (HA-LIP5, Myc-SKD1, or HA-LIP5 and Myc-SKD1) were immunoprecipitated with anti-Myc or anti-HA antibodies. These immunoprecipitates were then separated by SDS-PAGE and immunoblotted with anti-HA or anti-Myc antibodies. The numbers on the left show the molecular masses of marker proteins in kilodaltons. (a) Samples were immunoprecipitated with anti-Myc antibody and immunoblotted with anti-HA (upper) or anti-Myc antibody (lower). (b) Samples were immunoprecipitated with anti-HA antibody and immunoblotted with anti-Myc (upper) or anti-HA antibody (lower).

A yeast two-hybrid assay was performed to determine whether ZmSKD1 could interact with NtLIP5 *in vitro*. Combinations of plasmids expressing bait protein BD-ZmSKD1 and prey protein AD-NtLIP5 were transformed into *AH109*, respectively. It was verified that expression of BD-ZmSKD1 and AD-NtLIP5 was not toxic for *AH109* yeast cells and that these fusion proteins did not bind or trans-activate the *lacZ* reporter gene non-specifically when expressed separately (data not shown). Co-transformation assays showed that independent yeast colonies containing pGBKT7-ZmSKD1and pGADT7-NtLIP5, as well as those containing pGADT7-T and pGBKT7-p53 (positive control), grew well on SD/−Ade/−His/−Leu/−Trp medium and turned blue in the *β*-galactosidase colony lift filter assay, indicating that there were strong interactions between ZmSKD1and NtLIP5. In contrast, no growth was observed for the negative controls (pGBKT7-ZmSKD1+pGADT7 or pGBKT7+pGADT7-NtLIP5) (Fig. 6A). This suggested that ZmSKD1 has the ability to interact with NtLIP5 *in vitro*.

The interaction between ZmSKD1 and NtLIP5 in *N.benthamiana* leaves was further confirmed by an immunoprecipitation assay. The cell extracts were immunopurified with anti-Myc antibody to pull down Myc-SKD1 protein, and then the immunoprecipitated products were fractionated via SDS-PAGE and detected with anti-HA antibody. As shown in Fig. 6B-a, the HA-LIP5 protein can only be detected in the mixture of Myc-SKD1 and HA-LIP5 extracts (upper). Likewise, Myc-SKD1 protein can be detected in the Myc-SKD1 extracts or the mixture of Myc-SKD1 and HA-LIP5 extracts using anti-Myc antibody, but not in the HA-LIP5 extracts (lower). To verify the interaction, we performed the same assay, replacing the anti-Myc antibody with anti-HA antibody, and then detected the results with an anti-Myc antibody. In this assay, the Myc-SKD1 protein could also be detected in the same sample (Fig. 6B-b, upper); Also, HA-LIP5 protein can be detected in the HA-LIP5 extracts or the mixture of Myc-SKD1 and HA-LIP5 extracts using anti-HA antibody, but not in the Myc-SKD1 extracts (lower) (Fig. 6B-b, lower). Both results indicated that a direct interaction occurred between the ZmSKD1 and NtLIP5 proteins extracted from transient expression in *N.benthamiana* by agroinfiltration.These results, together with above results confirm that ZmSKD1 can interact with NtLIP5 *in vitro* as well as *in planta*.

### Mapping of the Domain of ZmSKD1 Required for Interacting with NtLIP5

**Figure 7:**
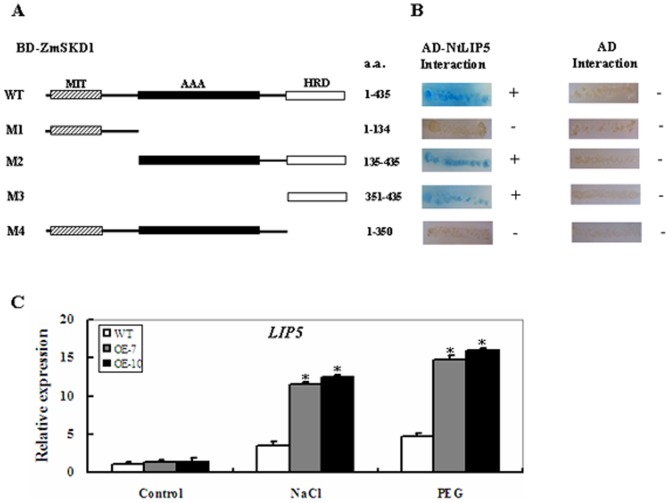
Mapping of the ZmSKD1 region involved in SKD1-LIP5 interaction and transcript levels of *NtLIP5* in responses to salt or PEG-induced water stress in transgenic and wild-type plants. **A** Schematic representation of wild type (WT) and mutant SKD1s in the study. M1 and M4 are C-terminal deletion mutants, and M2 and M3 are N-terminal deletion mutants. MIT marks microtubule-interacting and trafficking; AAA marks ATPase associated with a variety of cellular activities; HRD marks the α-helix-rich domain located at the C-terminus of SKD1. The numbers denote SKD1 amino acid positions. **B** The interaction between LIP5 or empty vector pGAD-T7 and SKD1 deletions in YTH assays. The ability of SKD1 truncations to interact with LIP5 or AD vector is indicated on the right (+, positive; −, negative). **C** *NtLIP5* transcript levels under salt or PEG-induced water stress in transgenic and wild-type plants. Four-week-old transgenic and wild-type tobacco plants were exposed to distilled water (control), 15% PEG6000 and 200 mM NaCl for 12 h, respectively, and leaf samples were used for real-time PCR analysis. *NtActin* was used as an internal control. For each treatment, the expression level under control conditions was defined as 1.0, and data represented means ± SE of three technical replicates. *t-test, with P<0.05.

Two functional domains of ZmSKD1 were identified; one is an N-terminal MIT domain (residues 7 to 73) and the other is a central AAA domain (residues 135 to 298) (Figs. 1A, 7A). In addition, a α-helix-rich domain (HRD) in the C-terminus (residues 351 to 435) was found in the ZmSKD1 protein by META Predict Protein Server analysis (http://www.embl-heidelberg.de/predictprotein/) (Fig. 7A).

To examine the roles of these three domains in mediating the interaction between SKD1 and LIP5 and to determine further the domain necessary for SKD1-LIP5 interaction, we sequentially constructed a serial of truncation derivatives that express different structural elements of the SKD1. Homologous binding capabilities between these truncated mutants and NtLIP5 were investigated via the YTH assay. Schematic representation of the different SKD1 truncations is shown in Fig. 7A. Firstly, the interactions between NtLIP5 and M1 (1–134) (the N-terminal region of 134 residues of ZmSKD1, Fig. 7A) or M2 (135–435) (the C-terminal region of 301 residues of ZmSKD1, Fig. 7A) were examined. As for wild type SKD1, M2 (135–435) was able to interact with the NtLIP5 (Fig. 7B). However, no clear yeast colonies growth was observed for M1 (1–134) (Fig. 7B), suggesting the C-terminal region containing AAA and HRD domains is responsible for SKD1-LIP5 interaction. Then, a deletion mutant M3 (351–435) was made by amplifying the fragment containing the last 85 residues from the C-terminus of SKD1, in which only the HRD was present (Fig. 7A).YTH results also showed a strong interaction between M3 and the NtLIP5 (Fig. 7B). Finally, the M4 mutant (1–350), a deletion of this HRD domain alone in the YTH assays led to the loss of interaction between M4 and the NtLIP5 (Fig. 7B). Also, control experiments included pGADT7 only with WT, M1, M2, M3, or M4 showed no positive interactions (Fig. 7B).Collectively, these results demonstrate that the C-terminal domain (residues 351–435) of ZmSKD1 is required for its interaction with NtLIP5.

### Changes in *LIP5* Gene Expression in *ZmSKD1* Transgenic Plants During Salt or Drought Stress

To further look at the changes in *LIP5* gene expression levels during salt or drought stress, transcript levels of *NtLIP5* were monitored in the wild-type and OE plants by qRT-PCR. After 12 h, the abundance of *NtLIP5* transcripts displayed differential patterns between the wild-type and OE lines. Under salt or PEG-induced water stress, compared with controls, the transcript levels of *NtLIP5* were significantly up-regulated in both WT and OE plants, but the magnitudes of increase in *NtLIP5* transcripts were differential (Fig. 7C). Under 200 mM NaCl stress, WT showed a 2.5-fold increase, while the two OE lines exhibited 7.5 and 8-fold increases, respectively (Fig. 7C). Similarly, under 15% PEG stress, the transcription levels of *NtLIP5* were significantly up-regulated in both OE lines (about 10-fold increase for both lines), while only 3.5-fold increase in the WT (Fig. 7C). In addition, no significant changes in *NtLIP5* transcripts were detected between WT and OE lines under control conditions (Fig. 7C). The results further indicate that *LIP5* together with *ZmSKD1* responds to salt or drought stress in transgenic plants.

## Discussion

Environmental stresses such as drought and high salinity have drastic effects on both biomass and grain yields of crops worldwide. However, the molecular mechanisms underlying AAA-type proteins-mediated ESCRT pathway involved in response to salt or drought stress in plants are largely unknown. In this study, we investigated physiological roles of a putative AAA-type ATPase SKD1 from maize and its possible function mechanisms using several complementary approaches. Our genetic evidence has demonstrated that ZmSKD1 can interact with NtLIP5 and its over-expression enhances not only salt but drought stress tolerance in transgenic plants. To the best of our knowledge, this is the first *SKD1* gene from monocot plants to be functionally characterized, in which the novel function for *SKD1* in drought has never been reported before in higher plants.

### *SKD1* is Conserved in Yeast, Plants and Mammals

Like known yeast, plant and mammal SKD1s, ZmSKD1 has higher sequence identities (52–93%) and two typical structural domains (Fig. 1A). From this, it can be reasonably concluded that the ZmSKD1 cDNA clone encodes maize SKD1 isoform. Both *Arabidopsis* and rice (*Oryza sativa*) contain one copy of the *SKD1*gene in their genomes [8]. A database search revealed that *ZmSKD1* is also encoded by a single-copy gene and located on chromosome 3 in maize genome. This indicates that *SKD1* is evolutionarily conserved and may be functionally important even essential in plant species. This view can be supported by the fact that over-expression of ATPase-deficient SKD1 caused a lethal phenotype in *Arabidopsis*, but not in yeast or mammalian cells [8].

### The Effects of *ZmSKD1* on Salt or Drought Stress Tolerance Might Be Specific in Plants

*ZmSKD1* was found to be highly expressed in aerial tissues of maize, and its highest expression level occurred in leaves (Fig. 2A), indicating this gene may be constitutively expressed during both vegetative and reproductive growth. This is consistent with the expression potential of *AtSKD1* as determined in *Arabidopsis* by transcript and protein levels analyses [8]. Previous studies have shown that SKD1 orthologs from *Arabidopsis* (*AtSKD1*) and ice plant (*McSKD1*) were induced by salt stress [16], [17], but no drought-induced expression of both genes was reported. Compared to this expression pattern, *ZmSKD1* is significantly affected not only by salt, but by drought stress in maize (Fig. 2B, 2C). Genetic evidence further proved that ZmSKD1 OE lines showed enhanced salt and drought tolerance in tobacco (Figs. 3, 4). These results indicate that function divergence of the *SKD1* orthologs may occur in the genomes of *Arabidopsis* and maize, although these genes have high similarities in sequences. In addition, the effects of *ZmSKD1* on heat or cold stress were tested using transgenic tobacco lines, but no significant enhancing effects have been found at present (data not shown); demonstrating that the ZmSKD1's effect on tobacco stress tolerance could be specific. Based on these results, it can be speculated that *ZmSKD1* might participate in salt and drought stress responses and plays a positive role in both stresses.

### The Possible Involvement of *ZmSKD1* in ROS Levels Modulation in Transgenic Plants During Salt and Drought Stresses

Proper ROS levels are critical for tolerance to abiotic stress in plants. In this study, both OE and WT leaves showed increased DAB staining within 1 h of NaCl or PEG stress (Fig. 5A, 5B); showing that *ZmSKD1* transgenic plants accumulated more H_2_O_2_ than the WT at early stage of stresses. After 2 h, OE plants showed less staining than the WT in DAB or NBT staining; moreover, the contrasting DAB staining was much clear between OE and WT leaves in the remaining of the period; the *ZmSKD1* transgenic plants accumulated less ROS than WT (Fig. 5). This observation indicated that the more H_2_O_2_ in transgenic plants at early stage might act as early signaling molecules to activate downstream ROS-scavenging or stress-responsive gene expression promptly, resulting in less toxic levels of ROS accumulations in subsequent stress period. In addition, there are fluctuations in both H_2_O_2_ and O_2_^−^ levels, especially in PEG-treated wild-type plants stained with DAB, and in salt-treated wild-type and OE plants stained with NBT (Fig. 5A, 5C). The fluctuations in ROS levels may indicate the complex regulating mechanisms of ROS's involvement during *ZmSKD1*-mediated drought or salt tolerance in plants. Thus, it could be speculated that *ZmSKD1* probably modulates ROS levels by more efficient removal or a delay in sensing or transducing the stress signals. Further work is needed to dissect the involvement of *ZmSKD1* in the ROS signaling network using microarray analysis in *ZmSKD1*-modified plants under drought or salt stress.

### ZmSKD1 might Interact with LIP5 in Transgenic Plants During Salt or Drought Stress

Database search has revealed that one *LIP5* homolog exists in the maize genome. It encodes a predicted protein of 477 amino acids, which is 51.7% and 49.3% identical to NtLIP5 and AtLIP5, respectively (Data not shown). This indicates that *LIP5* homologues have high similarities in sequences. The AtSKD1 can interact with AtLIP5 in yeast and plants [8]; also, the interaction between ZmSKD1 and NtLIP5 was confirmed by Y2H and immuno-precipitation (in this study); indicating that the LIP5 homologs are conserved in function. Thus it could be speculated that ZmSKD1 might interact with maize LIP5.

The *Arabidopsis* mutant *atlip5* plants are viable and show no phenotypic alterations under normal growth conditions, suggesting that basal SKD1 ATPase activity is sufficient for plant development and growth [8]. However, no further investigations have been reported for plant *LIP5* in response to salt or drought stress. In our study, we have found that ZmSKD1 is capable of interacting with NtLIP5 by its α-helix-rich domain in the C-terminus (Fig. 7A); moreover, the induced expression pattern of *NtLIP5* showed a resemblance to that of *ZmSKD1* under salt and drought stresses (Figs. 2A, 2B; 7C). More importantly, the transcript levels of *LIP5* in transgenic plants had greater magnitudes of increase than those in WT plants under salt or drought stress; suggesting that the increased LIP5 abundance may contribute to ZmSKD1 activity enhancement, resulting in increased endurance of transgenic plants under stress conditions. This notion could be proved by the evidence that *Arabidopsis* LIP5 acts as a positive regulator of SKD1 by increasing its ATPase activity by 4∼5 folds *in vitro* [8]. Although the roles of LIP5 in plant salt or drought tolerance have not been established, the expression patterns indicate that LIP5 might play a positive role in salt or drought tolerance by interacting with SKD1. The true roles of the *LIP5* gene under salt or drought stress need to be fully explored using transgenic plants in future studies.

Besides LIP5, AtSKD1 has been characterized to interact with ESCRT-III components and putative ESCRT-III-associated proteins on MVBs in *Arabidopsis* [13], [18], [19], [20]. Therefore, it will be interesting to compare possible mechanisms of SKD1's involvement in the ESCRT pathway under stress conditions between the model plant *Arabidopsis* and crop plants.

### SKD1-mediated ESCRT Machinery may Be Involved in Cellular Responses to Environmental Stress

The AAA ATPase SKD1 exploits the energy from ATP hydrolysis to induce conformational changes in other proteins or protein complexes causing unfolding or dissociation of substrate proteins [3]. It has been reported that the yeast *vps4/skd1* mutant showed cadmium sensitivity; suggesting that the ESCRT machinery is specifically involved in targeting of vacuolar proteins responsible for cadmium detoxification [21]. A microarray analysis on *AtSKD1*-knockdown *Arabidopsis* mutant under salt stress has shown that expressions of one stress-related kinase, several salt-induced transcription factors and one auxin efflux transporter were altered [14]. Moreover, the *SKD1*-knockdown plants experienced a reduced salinity response and altered root development; indicating the importance of intracellular protein trafficking in both auxin-mediated plant growth and in maintaining ion homeostasis under salt stress [14]. These lines of evidence could demonstrate that SKD1-mediated ESCRT machinery may be involved in cellular responses to environmental stress.

In summary, these data have demonstrated that the monocot *ZmSKD1* could be involved in salt and drought responses and its over-expression enhances not only salt but drought stress tolerance. This study may facilitate our understanding of the biological roles of SKD1-mediated ESCRT pathway under stress conditions in higher plants and accelerate genetic improvement of crop plants tolerant to environmental stresses.

## Materials and Methods

### Plant Material and Stress Treatments

A maize inbred line, *Zea mays* cv. Zheng58, was used throughout this study. Tobacco plants (*Nicotiana tabacum* cv. Xanthi) were used for gene transformation. Plants were grown in a growth room as described previously [22]. Drought or salt stress in two-week-old seedlings was realized by replacing the water with PEG 6000 (10%, 15%, or 20%) and NaCl (100, 200, or 300 mM), respectively, and leaves were sampled at 0, 6, 12, 24, or 48 h for expression analysis as described by us [23].

### Cloning of *ZmSKD1* and Sequence Analysis

The salt-induced EST encoding a putative AAA protein was used to do BLAST (http://www.ncbi.nlm.nih.gov/) and mRNA sequences containing such an EST were downloaded for gene prediction. The gene is highly homologous to *AtSKD1*, and thus is named *ZmSKD1*. Two primers SKD1-F and SKD1-R (Table S1) were designed for amplifying the open reading frame (ORF) of *ZmSKD1*. The 1308 bp PCR product was verified by sequencing.

The primary structural analysis was performed using InterProScan (http: //www. ebi.ac.uk/InterProScan). The alignment of the deduced protein sequences and phylogenetic tree analyses were done by DNASTAR and MEGA 5.1, respectively, using standard parameters [24].

### Real-time PCR Analysis

Real-time PCR was used to determine the expression patterns of *ZmSKD1* in different organs and under stress conditions. The qRT-PCR was performed in triplicate with an IQ5 light cycler system (Bio-Rad) using SYBR Premix ExTaq II (Takara, Japan) with gene-specific primers SKD1-F1 and SKD1-R1 (Table S1), which produces a141-bp product. The maize *Ubiquitin* transcript was used as an internal control to quantify the relative transcript levels [23]. The relative level of gene expression was detected using the 2^−ΔΔC^_T_ method [25].

The expression of *NtLIP5* in transgenic plants was analyzed by real-time PCR with RNA samples isolated from four-week-old transgenic plants harvested after 12 h of distilled water (control), 15% PEG6000 and 200 mM NaCl treatments. PCR was performed with specific primers NtLIP5-F3 and NtLIP5-R3 (Table S1), which produces a 138-bp product. Amplification of tobacco *Actin* was used as an internal control as described by us previously [22]. For the entire qRT-PCR assay, three technical replicates were performed for each experiment and the expression of each gene was investigated in three biological replicates.

### Construction of Plant Expression Vectors and Development of Transgenic Tobacco Lines

The *ZmSKD1* coding sequence was amplified and introduced into the pART7 plasmid using primers SKD1-F2 with *Eco*RI restriction site (underlined) and SKD1-R2 with *Bam*HI restriction site (underlined) (Table S1) and was subsequently inserted downstream of the 35S promoter in the plasmid vector pART7. The resulting expression cassette containing the 35S promoter and *ZmSKD1* coding sequence was cut and inserted into the binary vector pART27 [22], producing the transformation construct pART27-35S-*ZmSKD1*. The binary construct was introduced into *Agrobacterium tumefaciens* strain LBA4404 and then transformed into tobacco via the leaf-disc method as described by us previously [22].

The kanamycin-resistant transgenic plantlets were confirmed by PCR with primers 35SP-F and SKD1-R3 (the forward primer 35SP-F is from CaMV 35S promoter sequence) (Table S1). The PCR-positive plantlets were transplanted into soil for growing in the growth room. The transgenic tobacco progenies were selected using antibiotics and maintained growth to set seeds until T_2_ generation. Six independent homozygous *ZmSKD1* over-expression (OE) transgenic lines (named OE-1, OE-2, OE-4, OE-5, OE-7, and OE-10) were developed. The expression of the transgene in the six lines was evaluated by qRT-PCR with gene-specific primers SKD1-F4 and SKD1-R4 (Table S1), which produces a 159-bp product.

### Analysis of *ZmSKD1*-overexpressing Tobacco for Salt and Drought Stress Tolerance

For salt stress tolerance analysis, two sets of experiments were designed. One is assayed for seedlings in the MS medium plates. Two-week-old wild-type (WT) and homozygous T_2_ transgenic seedlings were transferred to 1/2 MS medium supplemented with 0, 100, 200 or 300 mM NaCl for growing vertically, in the growth chamber. The phenotype of seedlings was photographed after 10 d of growth and the root length and fresh weight of seedlings were measured. The other is treated with the potted adult plants. Six-week-old WT and T_2_ transgenic seedlings grown in soil were watered with 0 (water) or 300 mM NaCl solution every other day for 4 weeks. The experiment was repeated at least three times. Survival rates (%) under salinity stress were determined as the number of visibly green plants after 4 weeks.

For drought tolerance analysis, four-week-old plants were subjected to progressive drought by withholding water until a nearly lethal effect of dehydration (about 2 weeks) was observed on wild-type plants. The drought stress experiment was performed at least three times. Three days after rewatering, the fresh weight and total chlorophyll content of treated plants were measured as described previously [26]. The remaining chlorophyll content in WT or OE plants after drought was expressed as a percentage, in which the chlorophyll content of each treated genotype was compared to its own control plants with the same age without drought stress.

### Histochemical Detection and Quantitative Determination of H_2_O_2_ and O_2_^−^ Production

Histochemical detection of H_2_O_2_ accumulation with 3, 3′- diaminobenzidine (DAB) staining was performed as described by us previously [21]. Detection of O_2_^−^ with nitro blue tetrazolium (NBT) was performed according to the method of Overmyer *et al.* [27]. Four-week-old WT and *ZmSKD1*-overexpressing tobacco plants were treated with distilled water (control), 200 mM NaCl or 15% PEG-6000 solutions, respectively, for 1, 2, 6, and 12 h, and incubated with 100 µg mL^−1^ DAB or NBT solution. Stained leaf samples were boiled in 97% (v/v) ethanol to remove chlorophyll and photographed directly using a Canon camera.

Wild-type and OE (OE-7) plants were exposed to 200 mM NaCl or 15% PEG-6000 for 1, 2, 6, and 12 h; after that, relative H_2_O_2_ contents in leaves were assayed according to the method of Xia *et al* [22], and relative O_2_^−^ levels in leaves were quantified as described previously [28].

### Yeast two-hybrid Assays

Using the yeast two-hybrid (YTH) system, wild-type and three truncated SKD1 constructs were tested for their interactions with NtLIP5. WT was designed for expressing full-length SKD1 (1–435), whereas four mutants M1–M4 were constructed for expressing truncated SKD1s. M1 (1–134), M2 (135–435) were constructed by deleting 301 and 134 residues from the C-terminal and N-terminal ends of wild type SKD1, respectively. M3 (351–435) was made by amplifying the fragment containing the last 85 residues from the C-terminal end of SKD1, in which only the α-helix-rich domain (HRD) was present. M4 (1–350) was constructed by deleting 85 residues from the C-terminal end of wild type SKD1. Coding sequences of the WT SKD1 and four deletion mutants were amplified as *EcoR*I/*Bam*HI fragments using the primers listed in Table S1, and then individually cloned into the yeast expression vector pGBKT7. The recombinant plasmid pGADT7-LIP5 was created as *Nde*I/*Bam*HI fragment into the yeast expression vector pGADT7 using a similar strategy. All clones derived from PCR products were verified by sequencing and the recombinant plasmids w ere confirmed by restriction analyses. YTH assays were performed using the Matchmaker GAL4 Two-Hybrid System 3 (Clontech) as described by us [29]. All treatments were repeated at least three times.

### Plasmids Construction, Agroinfiltration and Immunoprecipitation in *N. benthamiana* Plants

For the 35S::*Myc-SKD1* construct, a PCR-amplified fragment of *ZmSKD1* was inserted into *Xba*I/*Bam*HI-digested plant expression vector pSPY1-35S with a 6xc-Myc tag to give the construct 35S::*Myc-SKD1*. For the 35S::*HA-LIP5* construct, the *NtLIP5* fragment with *Xba*I/*Bam*HI sites was cloned into plant expression vector pSPY2-35S with a hemagglutinin (HA) tag to give the construct 35S::*HA-LIP5*. The primers used in the experiment were listed in Table S1. Both constructs were introduced into *Agrobacterium tumefaciens* strains GV3101 and recombinant clonies were cultured for agroinfiltration.

Co-infiltration in *N.benthamiana* leaves were performed as described by us [30]. For immunoprecipitation assays, the infiltrated parts of *N. benthamiana* leaves were harvested and extracted. Corresponding antibodies (HA or Myc) were added to the cell lysates. Immunoblot analysis was conducted using anti-Myc (Santa Cruz, 1∶1000) or anti-HA antibody (Santa Cruz, 1∶1000) as described previously [22], [31].

## Supporting Information

Table S1 **PCR primers used in this study.**
(DOC)
