# Supplementary material for: The Maize AAA-Type Protein SKD1 Confers Enhanced Salt and Drought Stress Tolerance in Transgenic Tobacco by Interacting with Lyst-Interacting Protein 5
Source: PLoS One. 2013 Jul 24;8(7):e69787. doi: 10.1371/journal.pone.0069787 (PMC3722157; doi:10.1371/journal.pone.0069787)
Supplement: Table S1 — PCR primers used in this study. (DOC) [file pone.0069787.s001.doc]

**Table S1** **PCR primers used in this study**

Note: Basic residues are indicated in bold.

| Primer name | Primer sequence^*^ | Use |
| --- | --- | --- |
| SKD1-F  SKD1-R  SKD1-F1  SKD1-R1  SKD1-F2  SKD1-R2  35SP-F SKD1-R3  SKD1-F4  SKD1-R4  SKD1 (1-435)-F  SKD1 (1-435)-R  M1(1-134)-R  M2(135-435)-F  M3(351-435)-F  M4(1-350)-R  NtLIP5-F  NtLIP5-R  NtLIP5-F1  NtLIP5-R1  SKD1-F5  SKD1-R2  NtLIP5-F2  NtLIP5-R1  NtLIP5-F3  NtLIP5-R3 | 5'- ATGTATAGCAA CTTCAAG -3'  5'-TCAGCCCTCCTCGCCGAAC-3'  5'-CCCCAGGAACAGGAAAGTCTTATTT-3'  5'-CCATTTGGAACAGATTCGCAACTAA-3'  5′- ACGAATTCATGTATAGCAACTTCAAG-3′  5′- AGGGATCCTCAGCCCTCCT CGCCGAAC-3′  5′- CCTACTCCAAAAATGTCAAAG-3′  5′-CACATGGCATCCACATG TC-3′  5′- TAACTGAAAGTGATTTCGAGAGC-3′  5′-ACATGGCATCCACATGTC- 3′  5'-ACGAATTCATGTATAGCAACTTCAAG-3'  5'-AGGGATCCTCAGCCCTCCTCGCCGAAC-3'  5'-AGGGATCCTCAAACGTCGTTCCACTTAATG-3'  5'-ACGAATTCATGGCCGGCCTCGAGAGCGCCAAG-3'  5'-TGGAATTCATGGTGCGCAAAACGCAGGAC-3'  5'-ACGGATCCTCAGGGTTCAAACAACACGTC-3'  5'-ACATGTCGAAGGAGAACGAAC-3'  5'-GATAATGGGGTTCAGCAGAT-3'  5'-ACCATATGTCGAAGGAGAACGAAC-3'  5'-AGAGGATCCTCAGCTGATGGGTTTGTT-3'  5′- CATCTAGAATGTATAGCAACTTCAAG-3′  5′- AGGGATCCTCAGCCCTCCT CGCCGAAC-3′  5'-TCTCTAGAATGTCGAAGGAGAACGAAC-3'  5'-AGAGGATCCTCAGCTGATGGGTTTGTT-3'  5'-GTTCCCTCCTTGTCTCCCTTATT-3'  5'-TCCCAGCTCGATCTTGTTTATCT-3' | Gene cloning of *ZmSKD1*  qRT-PCR for *ZmSKD1* expression  Construction of plant expression vectors  Verification of transgenic plants  Transcripts detection of transgenic plants  Construction of wild type and truncated ZmSKD1 expression vectors for Y2H    Gene cloning of *NtLIP5*  Construction of NtLIP5 in pGAD for Y2H  Construction of ZmSKD1 and NtLIP5 expression vectors for immuno-precipitation assays  qRT-PCR for *NtLIP5* expression |

^*^ The underlined nucleotides constitute *Eco*RI (GAATTC), *Nde*I (CATATG), *Bam*HI (GGATCC), or *Xba*I (TCTAGA) restriction enzyme digestion sites.
